# Dietary diversity and determinants of young adults in central China: A cross-sectional study from 2015 to 2020

**DOI:** 10.3389/fnut.2022.931107

**Published:** 2022-09-30

**Authors:** Yi Zhou, Jiangang Wang, Yinglong Duan, Xiaofei Luo, Ziyu Wan, Yating Luo, Ying Li, Yaqin Wang, Jianfei Xie

**Affiliations:** ^1^Department of Health Management Center, The Third Xiangya Hospital of Central South University, Changsha, China; ^2^Xiang Ya Nursing School, Central South University, Changsha, China; ^3^Department of Emergency, The Third Xiangya Hospital of Central South University, Changsha, China; ^4^Department of Nursing, The Third Xiangya Hospital of Central South University, Changsha, China

**Keywords:** diet survey, dietary diversity, eating habits, factor analysis, China

## Abstract

**Background:**

Early adulthood is a vulnerable period for improved nutrition at all phases of the life cycle. However, there is limited research on diversity information in young adults from middle-income countries undergoing an apparent nutritional transition. The purpose of this study was to explore dietary diversity and determinants among young adults aged 18–35 years in central China.

**Methods:**

From January 2015 to December 2020, a cross-sectional survey of 49,021 young adults in a health management center of central China was conducted through report and phone-assisted self-report for information. The outcome variable was the Dietary Diversity Score. Independent variables included age, sex, race, material status, education, BMI, taste preference, regular meals, midnight snacks, sugared beverage/coffee consumption, and smoking/drinking status. Multivariate logistic regression was performed.

**Results:**

Of 49,021 young adults, 38,374 (78.3%) reported insufficient dietary diversity, and 422 (0.9%) reported sufficient dietary diversity. Light taste preference [adjusted odds ratio (aOR) = 2.325; 95% CI: 1.779, 3.039] and those who had meals regularly (aOR = 1.241; 95% CI: 1.018, 1.513) and consumed coffee (aOR = 2.765; 95% CI: 2.257, 3.387) were more likely to be associated with sufficient dietary diversity. Midnight snacks (aOR = 0.728; 95% CI: 0.588, 0.901) and sugary beverages (aOR = 0.666; 95% CI: 0.535, 0.829) were less likely to be associated with sufficient dietary diversity. Higher BMI (aOR = 1.092; 95% CI: 1.061, 1.125) was associated with higher odds of sufficient dietary diversity. Additionally, participants who were 18–30 years old, with master or above degree and away from cigarette/alcohol were more likely to report better dietary diversity.

**Conclusion:**

Our results painted a less than ideal nutritional condition affecting young adults. High-fat/sugar/salt dietary practices can lead to low dietary diversity, while high dietary diversity might have adverse BMI outcomes in youth. This study highlighted the importance of increasing the diversity of healthy and selective food items before wide recommendation for dietary diversity.

## Introduction

Early adulthood is recognized as a vulnerable period; optimum nutrition is critical at this time because of growth in nutritional demands and important eating behaviors (1). Adequate nutrition in youth plays an important role in both present and long-term health. Hence, this phase is possibly the only opportunity for the catch-up nutrition needed to avoid the vicious intergenerational effect of malnourishment.

A high-quality diet consists of adequate intake of micronutrients; balanced protein, carbohydrate, and fat intake; and abstemious consumption of unhealthy foods (2). Dietary diversity is generally accepted as a key part of a high-quality diet because consuming various foods across and within different diet groups contributes to adequate levels of vital nutrients (3). Diet diversity indicators have been found to be prospective measurement tools, especially in developing countries, due to their simplicity of implementation and their potential for large-scale use, in contrast to other food-consumption indicators that collect complicated quantitative information (4). Low Healthy Diet Index score was found to be associated with obesity and other chronic diseases (5). International studies have consistently reported a lower-quality diet in younger age groups than in older age groups, who often have a higher-quality diet (6, 7). However, dietary intake and diversity information are almost invisible in young age groups.

To date, there is a narrow understanding of determinants of dietary diversity among young adults. Dietary practice has an important determination of many aspects of diet variety and a substantial influence on youth nutrients. Young adults are usually more likely to engage in unhealthy eating habits, such as common meal skipping (8), frequent fast food consumption and dining out (9), high added sugar intake (i.e., sugar added to beverages during production) (10), and inadequate consumption of fruit and vegetables (11), compared to other age groups. Moreover, China is undergoing an apparent nutritional transition related to rapid economic growth (12–14). The diet pattern of citizens has shifted from traditional food (low in fats, mainly composed of carbohydrates, vegetables, and few animal-based foods) to a western diet (high in fats, sugar, and refined foods) (15, 16). As a long-term unhealthy eating behavior, western diet has proved to be such an important factor associated with non-communicable diseases (17). Given this evidence, there is concern that youth, as a vulnerable population, might be placed at higher risk of suffering from micronutrient deficiencies (18). Furthermore, energy-dense foods give rise to annual weight gain (9, 19). Although previous studies had been conducted in China (Qinghai Plateau and Taiwan), the association between body mass index (BMI) and dietary diversity is still unclear, which may be ascribed to differences in the age of the enrolled population (20, 21).

However, little research has examined how current eating patterns influence dietary diversity in youth populations faced with an increasing variety of food choices. Given that dietary patterns shift as a result of urbanization and the easy accessibility of low-cost processed food in developing countries, it is necessary to understand the potential effect of sociodemographic and diet behavior factors on dietary diversity. Therefore, the objective of this study was to explore dietary diversity and determinants among young adults aged 18–35 years in central China across individual-level sociodemographic, dietary practice, and BMI status characteristics.

## Methods

### Sample and data

Data for this article came from a cross-sectional survey conducted in a health management center of a general tertiary hospital located in central China between 1 January 2015 and 31 December 2020. This survey focused on young adults, so the inclusion criteria was aged from 18 to 35 years and participated in the survey voluntarily, and 49,648 young adults remained enrolled in after the selection process. Before young adults underwent physical examination, trained interviewers from general tertiary hospitals provided general instructions for this study and invited subjects to participate in the investigation. Interviewers then used structured questionnaires (pre-coded) to record the sociodemographic characteristics and dietary practices of the subjects. Physical examination was performed by trained professionals under standard procedures and by standardized instruments for BMI collection. The food consumption information was gathered using 24-h dietary recalls for three straight days (2 and 1 weekend day), collected by interviewers by phone after physical examination. Altogether, 49,021 individuals completed the survey, for a response rate of 98.7%.

### Sample size

Our main guideline was the review of Charan and Biswas (22), in which the sample size of cross sectional surveys is considered to be calculated through Z${}_{1-\text{α}/2}^{2}$p(1–p)/d^2^. In this formula, Z_1−α/2_ presents standard normal variate, p is expected proportion in population, and d is absolute error/precision. Confidence interval is set as 95% in this study, wherein Z_1−α/2_ = 1.96. Based on previous large study (23), the estimated proportion of low DDS in Chinese adults is not more than 55%, and the absolute error/precision is assumed to be 0.03. Therefore, this study has to contain 1,057 subjects at least. In order to decrease bias effectively and obtain information profoundly, more young adults were expected to be included.

### Ethical clearance

This study was approved by the Institutional Review Board (IRB) of the general tertiary hospital. All procedures followed the Declaration of Helsinki, and all essential permissions were obtained from the government and health commission. All young adults participating in this survey completely understood the purpose and agreed to participate in the investigation.

### Outcome variable

The outcome variable was the Dietary Diversity Score (i.e., DDS), which is based on the Chinese Dietary Guidelines. It was defined as the number of food groups consumed over 3 days based on the 24-h dietary recalls. All food items were classified into nine groups: grains (tubers, cereals, and roots), vegetables, fruits, meat (pork, beef, poultry, and organs), beans (beans, nuts, and seeds), eggs, fish (seafood, freshwater fish, and aquatic products), dairy (milk and products), and oil (animal and vegetable oil). If a participant consumed any food from the abovementioned categories, they would receive one point in the corresponding food category. Otherwise, they would be scored zero (such as sugar beverages, coffee, tobacco, and alcohol). Consuming different foods from the same category did not count repeatedly. The total score was the sum of nine food groups, and the maximum score could reach nine points. For this study, DDS was categorized into three degrees [insufficient DDS (1–3 points), moderate DDS (4–6 points), and sufficient DDS (7–9 points)].

### Independent variables

The independent variables included sex, age, race, material status, education, taste preference (24), regular meals, midnight snacks, sugary beverages, coffee, smoking/drinking status, and BMI. Body mass index (BMI) was calculated as weight (kg) divided by height squared (m^2^). Weight was measured to the nearest 0.1 kg (with light clothes on flat ground), and height was measured to the nearest 0.1 cm (without shoes). Supporting details of other independent variables were exhibited in Table 1.

**Table 1:** Specifics of questionnaire.

**Variables**	**Statement**
**1. Demographics**	
Sex	(1) What is your physiologic sex?
	A. Male
	B. Female
Age	(2) Which is your interval of age this year?
	A. 18–25 years old
	B. 26–30 years old
	C. 31–35 years old
Race	(3) What is your race?
	A. Minority (fifty-five kinds of ethnic minorities)
	B. Han nationality
Material status	(4) What is your current marital relationship?
	A. Married or common law marriage
	B. Single
	C. Divorced or widowed
Education	(5) What is your highest level of education?
	A. HS or lower (middle school, elementary school etc.)
	B. Technical school
	C. HND/Bachelor
	D. Master or above (doctor, post doctor etc.)
**2. Dietary Behaviors**	
Taste preference	(6) In general, which of the following describe most of the foods that you like?
	A. Heavy [salty (halogen/pickled products, the most common local delicacies locally), spicy (fresh peppers, dried chilies, chili sauce, or pepper oil) or fatty foods (fried foods or sweets) etc.]
	B. Light (unsalted foods, fruits, or vegetables etc.)
	C. Arbitrary taste (neither dislike nor like any of the taste above)
Regular meals	(7) Are you usually able to eat three meals on time and not snacks for meals?
	A. Yes
	B. No
Midnight snacks	(8) Do you often intake energy-dense and sugar sweetened snacks between 21:00 and 06:00?
	A. Yes
	B. No
Sugary beverages	(9) Do you drink sugared beverages?
	A. Yes (sugared fruit/milk drinks; sugared or “no-cal” cola; other soft drinks)
	B. No
Coffee	(10) Do you drink coffee, not coffee beverages?
	A. Yes (refers in particular to those without processing or mostly physical processes)
	B. No
**3. Life styles**	
Smoker	(11) Which of the following best describes your smoking status?
	A. Never
	B. Former (quit smoking for more than 1 year)
	C. Passive (more than 15 min a day and more than 1 day a week)
	D. Current (continued smoking for more than 1 year)
Drinker	(12) Which of the following best describes your drinking status?
	A. Never
	B. Former (quit alcohol for more than 1 year)
	C. Current (have 1 or more alcoholic drinks per week)

HND, Higher National Diploma; HS, high school.

### Statistical analysis

Multinomial logistic regressions were conducted to assess the associations between the DDS and the identified independent variables. Unadjusted and adjusted models were conducted. Bivariate association of the outcome variable with any of the independent variables was first examined using Pearson χ^2^ tests or ANOVA, and those that were associated with the outcome variable were included in adjusted models. Only the test of difference across all categories was provided, but 95% confidence intervals were also presented to allow consideration of more nuanced differences. Missing data of continuous variables were filled using the variable's mean. The significance level was set at *p* = 0.05 for the analyses. All statistical analyses were carried out using SPSS, version 25.0 for Windows (IBM Corp, Armonk, New York) and accounted for features of the survey design.

## Results

### Characteristics of the sample

Sociodemographic information is displayed by DDS in Table 2, which represents the population of 18–35 years old among central China. The sample included 49,021 young adults who participated in and responded to questions about dietary diversity, with a response rate of 98.7%. The proportion of respondents in each dietary diversity category was approximately equally distributed by gender. The majority of the participants were over 25 years old (84.6%), Han nationality (95.4%), married (63.8%), with a higher national diploma or bachelor's degree (78.3%), never-smoker (72.2%), or never-drinker (72.9%). The mean BMI of the participants was 22.72 (±3.48) kg/m^2^. Of note, the total percentages of participants who reported insufficient dietary diversity (78.3%) were much higher than any other.

**Table 2:** Demographic characteristics of samples across the three dimensions of dietary diversity score (*n* = 49,021).

**Variables**	**Overall**	**Insufficient**	**Moderate**	**Sufficient**	***χ^2^/F***	***p*-value**
		**(*n* = 38,374) *N* (%)**	**(*n* = 10,225) *N* (%)**	**(*n* = 422) *N* (%)**		
**Sex**					31.628	< 0.000
Male	22,800 (46.5)	18,101 (79.4)	4,504 (19.7)	195 (0.9)		
Female	26,221 (53.5)	20,273 (77.3)	5,721 (21.8)	227 (0.9)		
**Age (years)**					50.028	< 0.000
18–25	7,548 (15.4)	5,860 (77.7)	1,618 (21.4)	70 (0.9)		
26–30	21,289 (43.4)	16,414 (77.1)	4,664 (21.9)	211 (1.0)		
31–35	20,184 (41.2)	16,100 (79.8)	3,943 (19.5)	141 (0.7)		
**Race**					11.478	0.003
Minority	2,279 (4.6)	1,719 (75.4)	537 (23.6)	23 (1.0)		
Han nationality	46,742 (95.4)	36,655 (78.4)	9,688 (20.7)	399 (0.9)		
**Material status**					46.255	< 0.000
Married	31,277 (63.8)	24,779 (79.2)	6,241 (20.0)	257 (0.8)		
Single	17,218 (35.1)	13,183 (76.6)	3,874 (22.5)	161 (0.9)		
Divorced/widowed	526 (1.1)	412 (78.3)	110 (20.9)	4 (0.8)		
**Education**					544.034	< 0.000
HS or lower	1577 (3.2)	1,368 (86.7)	206 (13.1)	3 (0.2)		
Technical school	1,389 (2.8)	1,208 (87.0)	175 (12.6)	6 (0.4)		
HND/Bachelor	38,377 (78.3)	30,487 (79.4)	7,593 (19.8)	297 (0.8)		
Master or above	7,678 (15.7)	5,311 (69.2)	2,251 (29.3)	116 (1.5)		
**Taste preference**					194.938	< 0.000
Light	14,738 (30.0)	11,019 (74.8)	3,539 (24.0)	180 (1.2)		
Arbitrary	18,563 (37.9)	14,616 (78.7)	3,794 (20.5)	153 (0.8)		
Heavy	15,720 (32.1)	12,739 (81.0)	2,892 (18.4)	89 (0.6)		
**Regular three meals**					52.807	< 0.000
Yes	17,027 (34.7)	13,017 (76.4)	3,840 (22.5)	170 (1.0)		
No	31,994 (65.3)	25,357 (79.2)	6,385 (20.0)	252 (0.8)		
**Midnight snacks**					40.280	< 0.000
Yes	31,261 (63.8)	24,721 (79.1)	6,308 (20.2)	232 (0.7)		
No	17,760 (36.2)	13,653 (76.9)	3,917 (22.0)	190 (1.1)		
**Sugared beverages**					29.240	< 0.000
Yes	34,238 (69.8)	27,016 (78.9)	6,952 (20.3)	270 (0.8)		
No	14,783 (30.2)	11,358 (76.8)	3,273 (22.2)	152 (1.0)		
**Coffee**					196.355	< 0.000
Yes	17,373 (35.4)	13,064 (75.2)	4,073 (23.4)	236 (1.4)		
No	31,648 (64.6)	25,310 (80.0)	6,152 (19.4)	186 (0.6)		
**Smoker**					125.297	< 0.000
Never	35,400 (72.2)	27,404 (77.4)	7,678 (21.7)	318 (0.9)		
Former	980 (2.0)	733 (74.8)	231 (23.6)	16 (1.6)		
Passive	2,901 (5.9)	2,222 (76.6)	651 (22.4)	28 (1.0)		
Current	9,740 (19.9)	8,015 (82.3)	1,665 (17.1)	60 (0.6)		
**Drinker**					28.620	< 0.000
Never	35,748 (72.9)	27,933 (78.2)	7,517 (21.0)	298 (0.8)		
Former	377 (0.8)	278 (73.7)	87 (23.1)	12 (3.2)		
Current	12,896 (26.3)	10,163 (78.8)	2,621 (20.3)	112 (0.9)		
BMI (Mean ± SD)	22.72 ± 3.48	22.63 ± 3.46	23.02 ± 3.53	23.35 ± 3.55	58.619	< 0.000

BMI, body mass index; HND, Higher National Diploma; HS, high school.

Bivariate analyses of dietary diversity and variables thought to be related were performed using chi-square tests. Table 2 shows that all variables were associated with three categories of dietary diversity (*p* < 0.05). The results of the eating behavior section showed that the percentage of insufficient dietary diversity was higher among individuals who showed a heavy taste preference (81.0%), ate three meals irregularly (79.2%), liked midnight snacks (79.1%) or sugared beverages (78.9%), and disliked coffee (80.0%).

### Dietary diversity assessment

As shown in Table 3, two categorical variables (race and material status) were not associated (at the *p* = 0.05 level) with DDS in the unadjusted models and were therefore excluded from the adjusted model. Each independent variable was adjusted in cooperation with other independent variables. Table 4 shows the results of the adjusted model examining the association between DDS and all independent variables.

**Table 3:** Unadjusted multinomial logistic regressions of dietary diversity on independent variables (*n* = 49,021).

**Variables**	**Moderate-DDS vs. insufficient-DDS**				**Sufficient-DDS vs. insufficient-DDS**			
	**OR**	**95% CI**		***p*-value**	**OR**	**95% CI**		***p*-value**
BMI	**1.061**	**1.054**	**1.069**	**0.000**	**1.092**	**1.060**	**1.125**	**0.000**
**Sex**
Male	**0.798**	**0.753**	**0.846**	**0.000**	0.881	0.688	1.128	0.316
Female	Reference				Reference			
**Age**
18–25	**1.236**	**1.141**	**1.338**	**0.000**	**2.068**	**1.463**	**2.923**	**0.000**
26–30	**1.184**	**1.124**	**1.247**	**0.000**	**1.705**	**1.356**	**2.143**	**0.000**
31–35	Reference				Reference			
**Race**
Minority	1.097	0.991	1.213	0.073	1.070	0.699	1.638	0.756
Han nationality	Reference				Reference			
**Material status**
Married	0.864	0.697	1.072	0.184	0.935	0.344	2.541	0.895
Single	0.961	0.771	1.197	0.722	0.875	0.317	2.416	0.797
Divorced/widowed	Reference				Reference			
**Education**
HS or lower	**0.391**	**0.334**	**0.458**	**0.000**	**0.116**	**0.037**	**0.368**	**0.000**
Technical school	**0.365**	**0.308**	**0.432**	**0.000**	**0.258**	**0.112**	**0.592**	**0.001**
HND/Bachelor	**0.596**	**0.563**	**0.631**	**0.000**	**0.469**	**0.375**	**0.586**	**0.000**
Master or above	Reference				Reference			
**Taste preference**
Light	**1.417**	**1.336**	**1.503**	**0.000**	**2.325**	**1.778**	**3.039**	**0.000**
Arbitrary	**1.155**	**1.094**	**1.221**	**0.000**	**1.532**	**1.175**	**1.996**	**0.002**
Heavy	Reference				Reference			
**Regular three meals**
Yes	**1.149**	**1.097**	**1.203**	**0.000**	1.239	1.016	1.511	0.034
No	Reference				Reference			
**Midnight snacks**
Yes	0.960	0.913	1.009	0.106	**0.728**	**0.588**	**0.902**	**0.004**
No	Reference				Reference			
**Sugared beverages**
Yes	**0.897**	**0.852**	**0.944**	**0.000**	**0.667**	**0.535**	**0.830**	**0.000**
No	Reference				Reference			
**Coffee**
Yes	**1.294**	**1.234**	**1.357**	**0.000**	**2.780**	**2.267**	**3.410**	**0.000**
No	Reference				Reference			
**Smoker**
Never	**1.206**	**1.124**	**1.295**	**0.000**	**1.444**	**1.048**	**1.990**	**0.025**
Former	**1.427**	**1.216**	**1.674**	**0.000**	**2.330**	**1.319**	**4.117**	**0.004**
Passive	**1.280**	**1.151**	**1.423**	**0.000**	1.541	0.970	2.447	0.067
Current	Reference				Reference			
**Drinking**
Never	**0.900**	**0.848**	**0.954**	**0.000**	0.782	0.607	1.007	0.057
Former	1.109	0.865	1.422	0.415	**3.186**	**1.705**	**5.952**	**0.000**
Current	Reference				Reference			

BMI, body mass index; CI, confidence interval; DDS, dietary diversity score; HND, Higher National Diploma; HS, high school; OR, odds ratio. Significant values are displayed in bold.

**Table 4:** Adjusted multinomial logistic regressions^a^ of dietary diversity on independent variables (*n* = 49,021).

**Variables**	**Moderate-DDS vs. insufficient-DDS**				**Sufficient-DDS vs. insufficient-DDS**			
	**aOR**	**95% CI**		***p-*** **value**	**aOR**	**95% CI**		***p*** **-value**
BMI	**1.061**	**1.053**	**1.068**	**0.000**	**1.092**	**1.061**	**1.125**	**0.000**
**Sex**	
Male	**0.801**	**0.755**	**0.849**	**0.000**	0.879	0.687	1.124	0.303
Female	Reference				Reference			
**Age**	
18–25	**1.333**	**1.245**	**1.428**	**0.000**	**1.969**	**1.458**	**2.659**	**0.000**
26–30	**1.220**	**1.162**	**1.282**	**0.000**	**1.671**	**1.343**	**2.079**	**0.000**
31–35	Reference				Reference			
**Education**
HS or lower	**0.385**	**0.329**	**0.450**	**0.000**	**0.117**	**0.037**	**0.370**	**0.000**
Technical school	**0.359**	**0.304**	**0.425**	**0.000**	**0.260**	**0.113**	**0.596**	**0.001**
HND/Bachelor	**0.592**	**0.560**	**0.627**	**0.000**	**0.469**	**0.375**	**0.587**	**0.000**
Master or above	Reference				Reference			
**Taste preference**	
Light	**1.418**	**1.337**	**1.504**	**0.000**	**2.325**	**1.779**	**3.039**	**0.000**
Arbitrary	**1.156**	**1.094**	**1.221**	**0.000**	**1.532**	**1.175**	**1.996**	**0.002**
Heavy	Reference				Reference			
**Regular three meals**	
Yes	**1.145**	**1.093**	**1.199**	**0.000**	**1.241**	**1.018**	**1.513**	**0.032**
No	Reference				Reference			
**Midnight snacks**	
Yes	0.962	0.915	1.012	0.131	**0.728**	**0.588**	**0.901**	**0.004**
No	Reference				Reference			
**Sugared beverages**	
Yes	**0.898**	**0.853**	**0.945**	**0.000**	**0.666**	**0.535**	**0.829**	**0.000**
No	Reference				Reference			
**Coffee**	
Yes	**1.307**	**1.247**	**1.370**	**0.000**	**2.765**	**2.257**	**3.387**	**0.000**
No	Reference				Reference			
**Smoker**	
Never	**1.208**	**1.125**	**1.297**	**0.000**	**1.443**	**1.047**	**1.988**	**0.025**
Former	**1.426**	**1.215**	**1.673**	**0.000**	**2.334**	**1.321**	**4.123**	**0.004**
Passive	**1.281**	**1.153**	**1.425**	**0.000**	1.542	0.970	2.449	0.067
Current	Reference				Reference			
**Drinking**	
Never	**0.899**	**0.848**	**0.954**	**0.000**	0.782	0.607	1.007	0.056
Former	1.106	0.863	1.419	0.426	**3.208**	**1.718**	**5.991**	**0.000**
Current	Reference				Reference			

aOR, adjusted odds ratio; BMI, body mass index; CI, confidence interval; DDS, dietary diversity score; HND, Higher National Diploma; HS, high school.

^a^Adjusted model: all variables in the table are included in the model and control for each other. Significant values are displayed in bold.

### DDS1 results: Demographics

Males had 0.801 times greater odds of moderate DDS [95% confidence interval (CI) = 0.755, 0.849; *p* < 0.001] but not sufficient DDS (*p* = 0.303) compared to their female counterparts. Compared to those aged 31–35 years old, those aged 18–25 had 1.333 times (95% CI = 1.245, 1.428; *p* < 0.001) and 1.969 times (95% CI = 1.458, 2.659; *p* < 0.001) greater odds of moderate and sufficient DDS, respectively. Compared to those whose highest-level education was master's degree or above, participants whose highest level of education was high school or lower had 0.385 times (95% CI = 0.329, 0.450; *p* < 0.001) and 0.117 times (95% CI = 0.037, 0.370; *p* < 0.001) greater odds of moderate and sufficient DDS.

### DDS2 results: Dietary behaviors

Participants who preferred light or arbitrary taste had 1.418- (95% CI = 1.337, 1.504; *p* < 0.001) and 1.156-times (95% CI = 1.094, 1.221; *p* < 0.001) greater odds of moderate DDS and 2.325- (95% CI = 1.779, 3.039; *p* < 0.001) and 1.532-times (95% CI = 1.175, 1.996; *p* = 0.002) greater odds of sufficient DDS vs. insufficient DDS, respectively. Those who ate three meals regularly were 1.145 times (95% CI = 1.093, 1.199; *p* < 0.001) more likely to have moderate DDS and 1.241 times (95% CI = 1.018, 1.513; *p* = 0.032) more likely to have sufficient DDS than their counterparts. Those with coffee consumption were 1.307 times (95% CI = 1.247, 1.370; *p* < 0.001) more likely to have moderate DDS and 2.765 times (95% CI = 2.257, 3.387; *p* < 0.001) more likely to have sufficient DDS than those with insufficient DDS. Those who ate midnight snacks were less likely to have sufficient DDS by 0.728 times (95% CI = 0.588, 0.901; *p* = 0.004), but not moderate DDS (*p* = 0.131). Those with sugared beverage consumption were 0.898 (95% CI = 0.853, 0.945; *p* < 0.001) and 0.666 times (95% CI = 0.535, 0.829; *p* < 0.001) less likely to have moderate DDS and sufficient DDS vs. insufficient DDS, respectively. Equally important, high BMI was associated with higher odds of moderate and sufficient DDS by 1.061 (95% CI = 1.053, 1.068; *p* < 0.001) and 1.092 times (95% CI = 1.061, 1.125; *p* < 0.001).

### DDS3 results: Life styles

Never smokers had 1.208 times (95% CI = 1.125, 1.297; *p* < 0.001) and 1.443 times (95% CI = 1.047, 1.988; *p* = 0.025) greater odds of moderate and sufficient DDS, respectively, than their smoker counterparts. Former smokers had 1.426 times (95% CI = 1.215, 1.673; *p* < 0.001) and 2.334 times (95% CI = 1.321, 4.123; *p* = 0.004) greater odds of moderate and sufficient DDS, respectively, than their smoker counterparts. Passive smokers had 1.281 times (95% CI = 1.153, 1.425; *p* < 0.001) greater odds of moderate DDS but not sufficient DDS (*p* = 0.067) than their smoker counterparts. Never drinkers had 0.899 times (95% CI = 0.848, 0.954; *p* < 0.001) greater odds of moderate DDS but not sufficient DDS (*p* = 0.056) than their drinker counterparts. Former drinkers had 3.208 times (95% CI = 1.718, 5.991; *p* < 0.001) greater odds of having a sufficient DDS but not a moderate DDS (*p* = 0.426) than their drinker counterparts.

## Discussion

To the best of our knowledge, this is the first study to explore determinants of dietary diversity using individual data from a large, representative young adult sample. Insufficient dietary diversity was found to be widespread in central China (78.3%), even far above the oldest old (55.7%) (25). The findings of this study show that participants with light taste preference who had meals regularly and consumed coffee were more likely to report sufficient dietary diversity. Midnight snacks and sugary beverages were found to be inversely associated with sufficient dietary diversity. Importantly, there was a significant positive association between BMI and dietary diversity. In addition, we found that being away from cigarettes/alcohol, 18–30 years old, and with master or above degree were associated with better dietary diversity.

Considering that lower dietary diversity might be a risk factor for health *via* malnutrition (26), identifying dietary determinants are of interest. Overall, we found that differences in dietary practice accounted for diet variety. Regular meals were associated with higher dietary diversity, which is expected. Young people might skip meals, especially breakfast, which may spontaneously increase nutritional vulnerability in young adulthood, thus leading to a “Snacker” dietary pattern (27). Snacker behavior, as a result of not feeling hunger in the morning, can potentially affect feelings of satiety (28), which can lead to worse dietary diversity throughout the day (29). Midnight snackers, who display disturbed regular meal consumption to some extent, were less likely to report sufficient dietary diversity in this study. This finding is similar to previous evidence, which proved that night eating is related to low diet quality in adolescents (30). In particular, late eaters had fewer daily servings of fruit and vegetables and consumed greater weekly servings of fast food/soda (31, 32). Nocturnal ingestion is consistently rich in carbohydrates but limited in dietary food, such as sugary foods/sweets, breads, cereal products and dairy products (33). Understandably, these palatable foods always have high sugar or high fat contents, which may tend to be on the other side of light taste. Supporting this hypothesis is the finding that participants with heavy taste were less likely to have better dietary diversity in our multivariate logistic regression analyses. The heavy taste pattern followed the high-fat/sugar/salt consumption system and had a poorer nutrient profile than other preferences (29). In particular, solid fat and added sugar are recognized sources of empty calories without nutritional value (34); overconsumption can drive energy intake above caloric requirements as well as also crowd out more nutrient-rich foods (35, 36), which could possibly explain our findings.

Interestingly, higher coffee consumption increased the likelihood of high dietary diversity in our samples, but sugared beverages showed the reverse pattern. Another study (37) found that both coffee and sugared beverages were associated with unsatisfactory dietary quality. According to a food classification based on the extent of industrial processing, coffee belongs to minimally processed foods, while sugared drinks are part of ultra-processed food products (38). The UK National Diet and Nutrition Survey found that fruit/vegetable, fiber and protein intake notably decreased when minimally processed foods decreased or ultra-processed food intake increased (39). This is not a permit to overindulge, but minimally processed coffee does have some unique advantages. That suggests the food processing should also be taken into consideration in response to healthier food choices (40).

Importantly, we found a positive relationship between BMI and dietary diversity, which was supported by prior literatures in various developing countries. A weight disorders survey among Iranian young population under the age of 18 suggested that the total DDS increasing a unit was associated with BMI z-score increasing 0.08 units, meanwhile, related to an increased risk of overweight, obesity or abdominal obesity (41). Sri Lankan researches in groups of adults over 18 years old also showed significant positive associations between dietary diversity and BMI, as well as the level of energy consumption (42, 43). In fact, it has been suggested that higher dietary diversity is associated with higher intake of total energy (particularly from fat and saturated fat) and is linked to obesity (44). A systematic review including 14 studies indicated that the relationship between diversity and adiposity depends on the healthy degree of eatables. Though variety of less healthy intake had a connection with greater adiposity, variety of all kinds of foods and healthy eating related to reduced risk of metabolic-related outcomes (45). For example, the intake of low energy-dense items (fruits and vegetables) contributes to dietary diversity and that higher diet diversity is linked to a lower risk of obesity (46). Another study assessing diversity scores exclusively for fruits/vegetables found that, although energy intake increased across the variety of fruits and vegetables, the mean BMI decreased (47). Briefly, a varied diet should be selective (e.g., fruits and vegetables) rather than absolute, despite the association between BMI and dietary diversity needs to be further researched in developing regions.

Based on findings from this study, never and former smokers had much better dietary diversity than their current-smoker peers, which is consistent with research in Western populations (48). Notably, the diet-smoking association found in diverse populations is not a culture-oriented phenomenon (49). There are reasons to believe that quitting smoking has implications in the implementation of an effective nutrition improvement program regardless of the culture and ethnicity of the youth population. We expected to find never drinkers to be associated with sufficient dietary diversity, but this trend was non-significant; only former drinkers tended to be predictive. Prior literature shows some substitution of alcohol for foods, which may suggest that restrictions on alcohol consumption may help to achieve better dietary diversity (50).

Additionally, the dietary diversity of young adults has been reported to be associated with sociodemographic characteristics. Consistent with prior report in southwest China (51) and Spain (52–54), males in this study were less likely to report sufficient dietary diversity. Although high DDS score of women in developed country has been considered to a whole higher global dietary quality in women, increased risk of general and central obesity has been reported among developing populations with higher DDS, which may suggest the potential strategies for promoting healthy diet in women living in developing countries require more attention. This study also demonstrated that young adults with master or above degree, females or males, were more likely to report better dietary diversity. Graduate and postgraduate groups have been proved to have high nutrition knowledge and healthy diet habits (55). Residents with an increase in dietary knowledge tend to consume more various foods (56), which may reflect the unique presentation of nutrition literacy and probably accounts for our result.

Although older age groups have been linked to better total diet variety (57), we observed that participants aged between 18 and 30 were more likely to have better dietary diversity than those who aged 31 years or over. All of these may be explainable when there existed some extent of home meals, no matter the frequent cookers were themselves (58) or other inhabitants (59). Cooking preparation behaviors has been found to appear in emerging adulthood instead of adolescence, and closely associated with better dietary quality in the 10-year longitudinal study. It is remarkable that quite a number of young participants may be with attribute “Snacker” and worse dietary diversity in our study, which can also be rationalized by home cooking. Snackers were mainly characterized by consuming co-processed foods (29). Evidence from Canada suggested that the decrease of ultra-processed products consumption is necessary, which composes any substantial improvement of diet (60). Adults who aged 18–23 and consumed snacks frequently have been shown to cook meals at home hardly and have limited nutritional targeting (61), which may be the reason for our opposite findings in DDS. This meant that efforts to encourage more home meals among young adults and allow them to make healthier diet choices.

## Limitations and strengths

We recognize that our study has several limitations. First, the causal relationships could not be determined, as this was a cross-sectional study. Second, a generally accepted limitation for all self-reported food intake data is that a proportion of individuals may alter their diet pattern or misreport (commonly underreport) the foods/beverages they consume (62). Third, only a restricted number of covariates were collected and analyzed, which may have excluded other important factors devoted to dietary diversity. Fourth, selection bias might have also impacted the estimation of results because non-responders may have had dissimilar dietary patterns than those who chose to respond. For example, young adults who perceive themselves as having “unhealthy” eating habits may have refused to participate. Lastly, larger sample sizes in different regions in China are needed in future studies.

Our study was based on a population and focused on vulnerable young adult populations, with a nearly 100% response rate. There have been limited previous research with such an extensive sampling about eating behavior relationships with dietary variety of young adults. Our study adds new evidence based on nutrition-related data from this vulnerable period in the context of a middle-income country.

## Conclusions

Although the large youth population plays a critical role as the next generation, these results painted a less than ideal nutritional condition affecting young adults. The findings of young adults indicated dietary diversity was lower in those with high-fat/sugar/salt eating behaviors and was higher in high BMI populations. This study highlighted the importance of improving the diversity of healthy and selective food items before wide recommendation for dietary diversity. As a public health issue, further researches are required to assess dietary diversity associations with known disparities experienced by youth with unhealthy eating behaviors.

## Data availability statement

The raw data supporting the conclusions of this article will be made available by the authors, without undue reservation.

## Ethics statement

The studies involving human participants were reviewed and approved by the Institutional Review Board of the Third Xiangya Hospital of Central South University (No. 2020-S587). The patients/participants provided their written informed consent to participate in this study. Written informed consent was obtained from the individual(s) for the publication of any potentially identifiable images or data included in this article.

## Author contributions

The study was designed by JW, YLi, YW, and JX. Data were collected by ZW and YLuo. Analyzed by YZ, YD, and XL. Data interpretation and manuscript preparation were undertaken by YZ, JW, and JX. All authors approved the final version of the paper.

## Funding

This work was supported by the Wisdom Accumulation and Talent Cultivation Project of The Third Xiangya Hospital of Central South University (Grant No. YX202006) and the Special Funding for the Construction of Innovative Provinces in Hunan (Grant No. 2020SK2091).

## Conflict of interest

The authors declare that the research was conducted in the absence of any commercial or financial relationships that could be construed as a potential conflict of interest.

## Publisher's note

All claims expressed in this article are solely those of the authors and do not necessarily represent those of their affiliated organizations, or those of the publisher, the editors and the reviewers. Any product that may be evaluated in this article, or claim that may be made by its manufacturer, is not guaranteed or endorsed by the publisher.
